# Effects of *in vitro* repaglinide supplementation on improving sperm motility parameters, viability, and DNA integrity in normozoospermic and asthenozoospermic men

**DOI:** 10.5935/1518-0557.20230038

**Published:** 2023

**Authors:** Eshrat Kalehoei, Mehri Azadbakht, Nazanin Karimi, Fatemeh Nowrouzi, Nasrin Jalilian, Farahnaz Keshavarzi

**Affiliations:** 1 Infertility Treatment Center of ACECR, Kermanshah, Iran; 2 Department of Biology, Faculty of Science, Razi University, Kermanshah, Iran

**Keywords:** repaglinide, human sperm, motility parameters, hyperactivation, DNA integrity, asthenozoospermic

## Abstract

**Objective:**

Human sperm motility and hyperactivation (HA) are induced by different factors such as intracellular calcium concentration. Repaglinide is an antidiabetic drug that, via the blocking of ATP-sensitive potassium channels (K-ATP channels), depolarization of the β-cell membrane, and opening of the voltage-gated calcium channels leads to an increase in intracellular calcium. The present study aimed to examine the effects of repaglinide on in vitro sperm motility parameters, viability, and DNA integrity in normozoospermic and asthenozoospermic men.

**Methods:**

Semen samples were collected from two groups of normozoospermic donors and asthenozoospermic patients. The samples were washed free of seminal plasma and then treated with medium alone (control) or with 100 nM and 1µM concentrations of repaglinide. After 1 h of incubation, percent sperm motility and hyperactivation were assessed; after 2 h of incubation, sperm viability and DNA fragmentation rate were evaluated by the Eosin-Y and acridine orange staining, respectively.

**Results:**

In both groups, repaglinide at a concentration of 100 nM and 1µM significantly improved percent sperm motility, hyperactivation, and vital sperms with normal DNA; in specimens from normozoospermic men, the 1µM concentration had a noticeable effect on progressive motility; in samples from asthenozoospermic men, the highest hyperactivation rate was seen at a concentration of 100 nM as compared with the 1µM concentration and controls (*p*<0.05).

**Conclusions:**

Our results suggest that repaglinide can improve sperm motility, hyperactivity, viability, and DNA integrity in both normozoospermic and asthenozoospermic men.

## INTRODUCTION

Fertilization is an essential step in natural fertility. For fertilization to occur, both gametes must undergo changes at specific times. These changes occur with the aid of specific biochemical and molecular agents that interact with the gametes (Anifandis *et al.*, 2014; Hernández-Falcó *et al.*, 2022). In mammals, sperm cannot fertilize the oocyte immediately after ejaculation and must first undergo capacitation. Capacitation is a complex process of functional biochemical and biophysical modification during which ejaculated spermatozoa, after passing through the female genital tract, become able to undergo acrosome reaction and fertilize the oocyte (Pitnick *et al.*, 2020; McPartlin *et al.*, 2009).

Most of the information about the different aspects of sperm capacitation has been obtained from *in vitro* studies. Sperm capacitation may occur in vitro with the use of laboratory media. Although there are few variations in the media used in the assisted reproduction of mammalian species, most contain compounds such as bicarbonate, calcium, and serum albumin (Yanagimachi, 1994; Nishimura *et al.*, 2004; Bianchi *et al.*, 2014). One of the functional events of capacitation is the development of a distinct motility pattern called hyperactivation, which is generally characterized by flagellar beating vigor, high amplitude head movements, and a nonlinear trajectory (Chang & Suarez, 2011).

After coitus, sperm must be able to migrate to microenvironments in the female reproductive tract to reach the oocyte. These events include an increase in sperm motility, hyperactivation, chemotaxis toward the oocyte, and finally acrosome reaction, which allows sperm to penetrate the oocyte. These physiological responses are triggered via the activation of sperm ion channels that occurs following a rise of sperm intracellular pH and Ca^2+^ in response to certain factors in the female reproductive tract (Suarez & Pacey, 2006; Darszon *et al.*, 2006).

The development of non-invasive and pharmacological treatment has been severely hindered by our limited understanding of cellular and molecular activity of the mature spermatozoa (Aitken & Henkel, 2011; Barratt *et al.*, 2011). In order to improve fertilization results, some have tried to understand sperm ion channels. Today, the identification of sperm ion channels using the whole-cell patch-clamp technique and optical methods to measure intracellular Ca^2+^ and pH in sperm cells has allowed the identification of several species-specific ion channels involved in the control of sperm activity and male fertility. These ion channels apparently differ from one species to another, and this contributes to our understanding of numerous unexplained cases of male infertility and the development new non-hormonal contraceptives. For example, the generation and characterization of CatSper knockout mice, by focusing on the production and regulation of calcium signals, can control sperm motility and hyperactivated motility in particular (Kirichok & Lishko, 2011; Lishko *et al.*, 2011; Alasmari *et al.*, 2013; Tamburrino *et al.*, 2014).

Poor sperm quality is a common reason for male infertility and may be associated with having a decreased sperm count (Oligozoospermia) or no sperm at all (Azoospermia). A significant portion of cases is caused by decreased sperm motility, also known as Asthenozoospermia (WHO, 2010). In bovine, murine, and human sperm, having an increased intracellular calcium level is essential to start and maintain hyperactivated motility. Therefore, abnormal motility may be ascribed to decreased cytoplasmic calcium levels (Dcunha *et al.*, 2022; Marquez *et al.*, 2007).

Antidiabetic drugs belonging to the class of sulfonylureas or meglitinide analogs such as repaglinide inhibit K-ATP channels and depolarize the cell membrane, and subsequently cause the opening of voltage-gated calcium channels leading to an increase in intracellular Ca^2+^ influx and insulin secretion from pancreatic β-cells (Ashcroft & Rorsman, 1989). Immunocytochemistry studies found K-ATP channel subunits in the sperm of several mammalian species (Acevedo *et al.*, 2006; Lybaert *et al.*, 2008). Based on this finding, one may hypothesize that drugs belonging to the class of sulfonylureas such as meglitinide analogs may have a beneficial role in modulating Ca^2+^ homeostasis. Therefore, considering the role of repaglinide in increasing intracellular calcium concentration, this study looked into the effects of repaglinide on sperm motility parameters, viability, and DNA integrity in normozoospermic and asthenozoospermic men.

## MATERIAL AND METHODS

### Materials

Chemicals were purchased from Origio, Denmark; repaglinide was purchased from the Farabi Corporation, Iran.

### Semen collection

This study was approved by the ACECR Infertility Treatment Center in Kermanshah, Iran, and by the ACECR Ethics Committee. Human semen samples from 60 donors were collected by masturbation after 2-5 days of sexual abstinence. After ejaculation, the samples were allowed to liquefy for 30 min at 37°C. Semen analysis was performed according to the standard criteria, which included semen volume, sperm count, motility, and morphology as defined by the World Health Organization (WHO, 2010). The donors were categorized as normozoospermic (motility >40%) or asthenozoospermic (motility <40% or progressive motility <32%).

### Preparation and incubation of sperm

After liquefaction, motile sperm from normozoospermic donors were retrieved from the samples after a double wash in sperm wash medium (3000 rpm for 5 minutes) and by employing the swim-up technique (using sperm wash medium supplemented with 2.5% HSA). After 1h of incubation, the supernatant containing motile spermatozoa was removed carefully and equally divided into aliquots. An aliquot without repaglinide was used as a control and for the experimental groups the medium was supplemented with 100 nM or 1 µM concentrations of repaglinide. The two repaglinide concentrations were selected from a previous study by Kalehoei & Azadbakht (2017). Dimethyl sulfoxide 0.1% (DMSO) was used to dissolve repaglinide as described by Fernandes *et al.* (2016). Sperm from asthenozoospermic patients was first double washed in sperm wash medium (3000 rpm for 5 minutes). The pellet was resuspended in medium without repaglinide and with different concentrations of repaglinide. Total motility, hyperactivity (HA), and percent viability, and DNA fragmentation were recorded at 1h and 2h post-incubation, respectively (37^o^C, 5% CO_2_) for all treatments according to the method described by Mukhopadhyay *et al.* (2010) and du Plessis *et al.* (2010) with some modifications.

### Assessment of motility and hyperactivity

**Figure 1:**
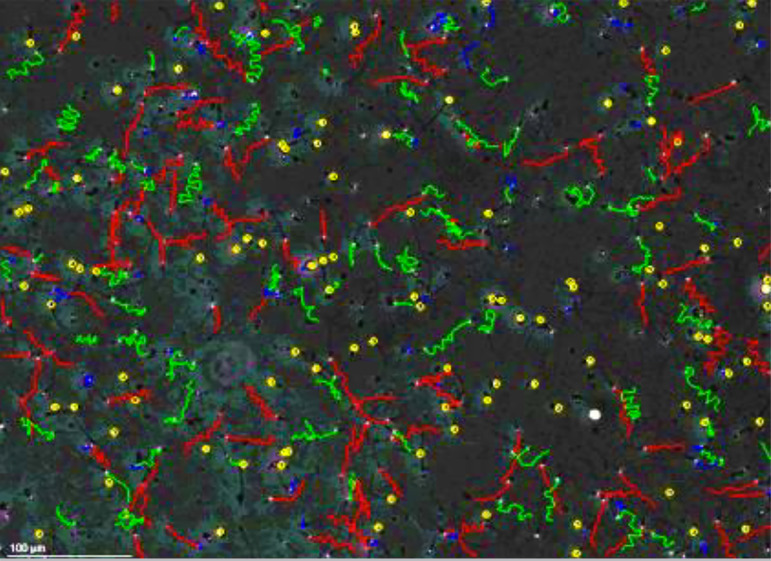
Image obtained from computer-assisted sperm analysis (CASA) (Scale bar: 100 µm).

Sperm motility was assessed based on the WHO standard criteria (WHO, 2010), which includes progressive motility (PR, spermatozoa moving actively, either linearly or in a broad circle, regardless of speed), non-progressive motility (NP, all other patterns of motility with an absence of progression), and total motility (PR + NP). The percentage of sperm motility was subjectively evaluated by two examiners using a light microscope (CX31; Olympus; Japan) at 400x magnification from a small drop of sperm suspension placed on a glass slide pre-heated at 37°C and covered with a 22 x 22 mm coverslip. Hyperactivated motility in each sample was assessed with the aid of computer-assisted sperm analysis (CASA). Five µl of semen samples were dropped into a sperm analysis chamber. At least 200 sperm were assessed randomly in 5-10 fields using CASA at 37°C (Figure 1) for sperm concentration, sperm motility, and different sperm movement characteristics such as curvilinear velocity (VCL), straight-line velocity (VSL), average path velocity (VAP), linearity(LIN= VSL/VCL), straightness (STR= VSL/VAP), wobble (Wob= VAP/VCL)), amplitude of lateral head displacement (ALH), and beat/cross-frequency (BCF). After 1h of incubation of motile sperm, the percentage of hyperactivated sperm (HA, %) was estimated manually based on the following threshold values: VCL ≥150 µm/s, ALH ≥7 µm and LIN ≤50%, all of which previously used in the definition of hyperactivated motility (Mortimer *et al.*, 1998).

### Viability assessment

A viability test is essential to differentiate dead sperm from immotile and live sperm. Eosin-Y staining (0.5% wt/vol) was performed at 0h and 2h post-incubation (37°C, 5% CO_2_) for all samples by mixing 1 ml of sperm suspension with 1 ml of Eosin-Y stain. Then, 10 µl of the sample was pipetted and placed on a microscope slide with a 22 × 22 mm coverslip and a total of 100-200 sperm were counted. The results were expressed by the percentage of unstained (viable) and nonviable (stained) sperm, according to WHO guidelines (mean percent viability remained above the lower reference value of 58%) (WHO, 2010).

### Sperm DNA fragmentation test

**Figure 2:**
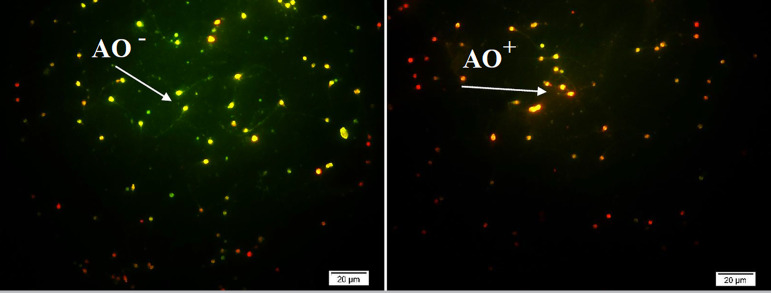
Assessment of sperm DNA fragmentation by Acridine Orange staining (Scale bar: 20 µm)

Different methods have been used to evaluate sperm DNA damage rate, and one of them is the Acridine Orange Test (AOT). In this method, smears of sperm samples are fixed in Carnoy’s fixative (methanol/glacial acetic acid, 3:1) for 2h; then the slide is dipped in acridine orange stain (0.19 mg/ml in McIlvain phosphate-citrate buffer, pH=4) for 5-10 minutes and rinsed with tap water. The percentage of damaged DNA and intact sperm is evaluated on a fluorescence microscope (Olympus, IX71; Japan). Sperm with normal DNA emits green fluorescence and damaged DNA emits orange/red fluorescence (Talebi *et al.*, 2012) (Figure 2).

AO -: **Green color shows sperm with normal DNA.**

AO +: **Red/Orange color shows sperm with damaged DNA.**

### Statistical analysis

In this study, results were expressed as means ± standard error of the mean (SEM). Analyses were performed on SPSS version 19 (SPSS Inc). Statistically significant differences between groups were assessed by ANOVA, post hoc, Tukey’s HSD test; statistical differences occurred when *p*<0.05.

## RESULTS

### Effect of repaglinide on sperm motility

**Figure 3:**
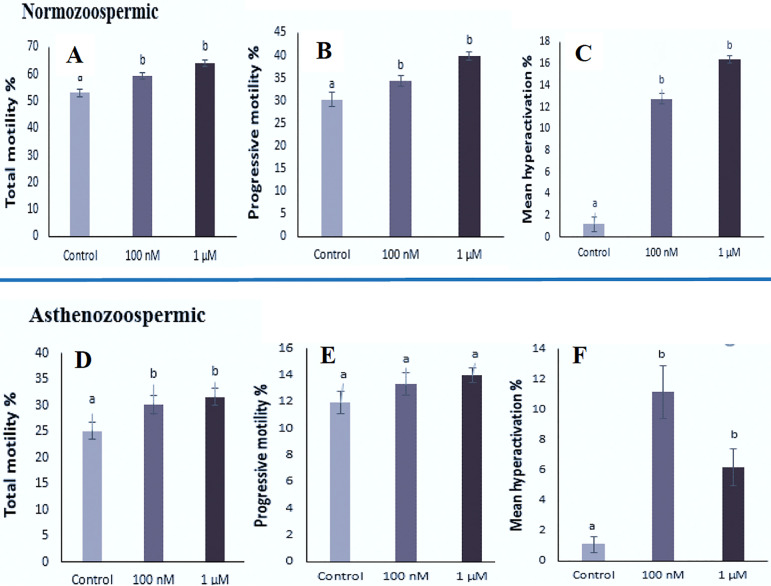
Effects of different concentrations of repaglinide (control, 100 nM and 1µM) on (A, D) Total motility; (B, E) Progressive motility; (C, F) Hyperactivity in normozoospermic and asthenozoospermic groups

Our study found significant differences in total motility after treatment with 100nM and 1µM concentrations of repaglinide in both normozoospermic and asthenozoospermic groups when compared to controls (Figure 3). Percent motility in normozoospermic samples from controls and specimens treated with 100nM and 1µM concentrations of repaglinide were respectively (53.15±1.33; 59.20±1.15; 64.10±1.13, *p*<0.05) and progressive motility were (30.30±1.50; 34.35±1.12; 39.95±0.97, *p*<0.05). The highest percent progressive motility was observed in the specimens treated with 1µM concentration of repaglinide. In the specimens from asthenozoospermic men, percentages of total motility were (25.10±1.67; 30.15±1.65; 31.60±1.58, *p*<0.05) and progressive motility were (11.95±0.85; 13.35±0.84; 14.00±0.57, *p*<0.05); there was no significant difference between controls and specimens treated with 100nM and 1µM concentrations of repaglinide.

Data were expressed as means ± SEM (%)

^a,b^ Indicates significant difference between the treatment and control groups (ANOVA test, *p*<0.05).

### Effect of repaglinide on sperm hyperactivity

Significant differences were observed in hyperactivity after treatment with 100nM and 1µM concentrations of repaglinide when compared with controls in both normozoospermic and asthenozoospermic specimens after 1h of incubation (Figure 3, Table 1). The results showed that in the normozoospermic group percent hyperactivated motility in controls and specimens treated with 100nM and 1µM concentrations of repaglinide were respectively 1.20±0.69; 12.75±0.50 and 16.40±0.36 (*p*<0.05); percent HA in specimens treated with 1µM concentration of repaglinide was higher than in the specimens treated with 100 nM of repaglinide and controls. In the asthenozoospermic group, percent hyperactivity was 1.1±0.53; 11.16±1.74 and 6.20±1.23 (*p*<0.05); the highest HA% belonged to the specimens treated with 100 nM concentration of repaglinide. Sperm movement parameters such as VCL in the specimens treated with repaglinide were significantly higher than controls in both the normozoospermic and asthenozoospermic groups (*p*<0.05).

**Table 1:** Demographics of recruited patients.

	Normozoospermic			Asthenozoospermic		
	Control	R 100 nM	R 1µM	Control	R 100 nM	R 1µM
VCL (µm/s)	114.79±2.38^a^	121.42±1.98^b^	124.55±2.04^b^	81.25±2.17^a^	94.33±1.76^b^	89.54±2.41^b^
VSL (µm/s)	58.13±2.19^a^	64.71±1.83^b^	67.34±1.45^b^	37.94±2.23^a^	48.56±2.11^b^	45.63±1.85^b^
VAP (µm/s)	59.43±1.77^a^	65.95±2.14^b^	66.74±1.68^b^	38.40±2.3^a^	49.78±2.51^b^	46.58±2.15^b^
LIN %	50.61±2.44^a^	53.27±2.31^a^	54.08±1.53^a^	46.61±2.06^a^	51.44±2.22^b^	50.96±1.53^a^
STR %	96.81±1.56^a^	97.11±2.05^a^	98.76±2.37^a^	97.42±2.51^a^	97.51. ±1.86^a^	97.22±2.15^a^
WOB %	51.65±2.11^a^	54.21±1.94^a^	53.49±2.42^a^	47.21±2.23^a^	52.14±1.72^a^	50.92. ±1.24^a^
ALH (µm/s)	2.26±1.58^a^	2.56±2.02^a^	2.97±2.14^b^	2.05±1.72^a^	2.34±2.23^a^	2.12±2.36^a^
BCF (Hz)	11.15±1.83^a^	12.57±2.31^a^	12.74±1.66^b^	8.55±1.35^a^	8.72±2.46^a^	8.40±1.87^a^
Hyperactive %	1.2±0.69^a^	12.75±0.50^b^	16.40±0.36^b^	1.1±0.53^a^	11.16±1.74^b^	6.20±1.23^b^

Data are expressed as means ± SEM.

a/b Values within columns with different superscripts are significant differences (ANOVA, *p*<0.05).

Control: without repaglinide; R _100 nM_: 100 nM concentration of repaglinide; R _1µM_: 1µM concentration of repaglinide.

VCL: Curvilinear velocity, VSL: Straight-line velocity, VAP: Average path velocity, LIN: Linearity, STR Straightness, Wob: Wobble, ALH: Amplitude of lateral head displacement, BCF: beat/cross-frequency.

### Evaluation of sperm viability

Longer incubation time causes viability decreases. At the start of the experiment (0h), sperm viability was 84% in the normozoospermic group; after 2h of incubation, percent sperm viability of controls and specimens treated with 100 nM and 1µM concentrations of repaglinide were 61%, 69%, and 72%, respectively. There was a significant difference between controls and specimens treated with repaglinide, but not between sperm treated with 100nM and 1µM concentrations of repaglinide (*p*>0.05). Percent viability of the asthenozoospermic group at 0h was 38%; after 2h of incubation, percent viability of controls and specimens treated with 100 nM and 1µM concentrations of repaglinide were 24%, 31%, and 33%, respectively (Table 2). Percent viability in controls was significantly lower than in specimens treated with repaglinide (*p*>0.05).

**Table 2:** Viability rate at 2h of incubation with repaglinide in normozoospermic and asthenozoospermic groups.

Groups	Normozoospermic		Asthenozoospermic	
	Viability (%)		Viability (%)	
	0h	2h	0h	2h
**Control**	84.16±0.24^a^	61.23±0.18^a^	38.47±0.06^a^	24.35±0.22^a^
R _100 nM_	83.12±0.30^a^	69.48±0.21^b^	37.22±0.12^a^	31.21±0.20^b^
R_1µM_	83.92±0.11^a^	72.54±0.32^b^	38.02±0.18^a^	33.56±0.12^b^

Data are expressed as means ± SEM.

a/b Values within columns with different superscripts are significant differences (ANOVA, *p*<0.05).

Control: without repaglinide; R _100 nM_: 100 nM concentration of repaglinide; R _1µM_: 1µM concentration of repaglinide.

### Assessment of sperm DNA fragmentation

After 2h of incubation, acridine orange staining results showed that in both normozoospermic and asthenozoospermic groups the percentage of green sperm (normal DNA) in the specimens treated with repaglinide (100 nM and 1µM concentrations) were higher than in controls. In the normozoospermic group, the percentages of orange/red sperm (damaged DNA) in controls and specimens treated with 100nM and 1µM concentrations of repaglinide were 18.21%, 11.34%, and 8.89%, respectively; in the asthenozoospermic group, the percentage of sperm with damaged DNA in controls and specimens treated with 100 nM and 1µM concentrations of repaglinide were 37.63%, 29.52%, and 26.48% (*p*>0.05). Comparisons between controls and specimens treated with repaglinide revealed a significantly higher rate of DNA damage in both normozoospermic and asthenozoospermic groups (Table 3).

**Table 3:** Sperm DNA fragmentation rate at 2 h of incubation with repaglinide in normozoospermic and asthenozoospermic groups.

Groups	Normozoospermic	Asthenozoospermic
	DNA fragmentation (%)	DNA fragmentation (%)
**Control**	18.21±0.28^a^	37.63 ±0.30^a^
R_100 nM_	11.34 ±0.32^b^	29.52±0.34^b^
R_1µM_	8.89±0.22^b^	26.48 ±0.32^b^

Data are expressed as means ± SEM.

a/b Values within columns with different superscripts are significant differences (ANOVA, *p*<0.05).

Control: without repaglinide; R _100 nM_: 100 nM concentration of repaglinide; R _1µM_: 1µM concentration of repaglinide.

## DISCUSSION

The present study showed that samples treated with repaglinide had significantly higher percent total motility, hyperactivity, and viability; DNA integrity rates were also significantly increased in normozoospermic and asthenozoospermic groups in comparison with controls.

Evaluation of human sperm motility characteristics such as hyperactivated motility in response to physiological stimuli can be useful to diagnose the fertility potential of human spermatozoa (Sukcharoen *et al.*, 1995). Ca^2+^ signaling plays a vital role in sperm activity control. In human sperm, depending on the source of calcium, Ca^2+^ signaling can be very compartmentalized to perform a specific function (Publicover *et al.*, 2007). The study by Kirichok *et al.* (2006) demonstrated that an important source of Ca^2+^ for hyperactivation is extracellular Ca^2+^ brought in by plasma membrane Ca^2+^ channels. In some cases, defects in calcium signaling leads to sperm dysfunction. Several clinical studies with infertile patients with oligozoospermia and teratozoospermia described the relationship between sperm function, progesterone, and the role of intracellular Ca^2+^ in sperm motility and acrosome reaction (Falsetti *et al.*, 1993; Oehninger *et al.*, 1994; Tesarik & Mendoza, 1992; Krausz *et al.*, 1996). The study by Espino *et al.* (2009) indicated that progesterone induces calcium signaling and that calcium intake in asthenozoospermic compared to normozoospermic men is much lower; therefore, the decreases in sperm motility and reproductive competency in these men may be associated with sperm calcium mobilization disruption. The study of human sperm showed that there are different channels such as potassium channels, which defects cause a low rate of fertilization and subfertility (Mansell *et al.*, 2014; Brown *et al.*, 2016). Data obtained from IVF samples showed that there is a correlation between in vitro fertilization rate and sperm hyperactivation. For example, the effect of 4-AP to increase intracellular Ca^2+^ and subsequently induce hyperactivity was significantly associated with IVF outcome, providing clear evidence about the biological role of Ca^2+-^signaling in human sperm (Lishko *et al.*, 2011; Strünker *et al.*, 2011).

One of the factors that lead to male infertility is asthenozoospermia. However, the development of pharmacological agents to improve sperm motility lacks effective screening platforms and knowledge of molecular targets. Nevertheless, significant progress has been recently made in the identification of compounds that may be used in the treatment of sperm dysfunction. Several studies using high-throughput screening (HTS) platforms have identified a large number of compounds that increase sperm activity. Interestingly, these drugs significantly enhanced calcium influx and motility in patient samples. However, further trials are required to examine the effectiveness of these drugs. In this regard, a pharmacological agent to improve sperm fertilization rate or decrease fertilizing potential (for male contraception) may provide a way to increase the success rate of conventional IUI and IVF methods (Martins da Silva *et al.*, 2017; McBrinn *et al.*, 2019; Gruber *et al.*, 2022; Alasmari, 2017).

Ion channels are important therapeutic targets for numerous disorders. Moreover, as some patients with defective Ca^2+^ storage exhibit low IVF rates, the development of effective and safe drugs designed to act as Ca^2+^ storage stimulants may be beneficial for this subgroup of individuals (Alasmari *et al.*, 2013). A study by Acevedo *et al.* (2006) using immunocytochemistry indicated that K-ATP channel subunits are present in adult mouse sperm and that the sulfonylurea receptor-2 subunit was immunolabelled mainly in the flagellum principal section. Lybaert *et al.* (2008) demonstrated that K-ATP channel subunits such as Kir6.2 and SUR2 are present in epididymal epithelial cells and in the spermatozoa of several mammalian species such as dogs, felines, cattle, and humans. Repaglinide is an antidiabetic drug that belongs to the meglitinide family; it decreases blood glucose levels by inducing the release of insulin in pancreatic islets (Alasmari *et al.*, 2013). Repaglinide acts by blocking K-ATP channels and depolarizing the bcell membrane. Therefore, it causes an increase in intracellular Ca^2+^ concentration by opening the voltage-gated calcium channels and inducing insulin secretion (Du *et al.*, 2006). According to these reports, drugs belonging to the class of sulfonylureas such as meglitinide analogs may have a beneficial role in modulating Ca^2+^ homeostasis.

Fernandes *et al.* (2016) showed that both inhibition and activation of K-ATP channels with drugs targeting K-ATP channels such as glibenclamide, 2, 4-dinitrophenol (DNP), and pinacidil are useful in maintaining Ca^2+^ homeostasis in oocytes in in vitro culture. Our research team recently indicated that the application of repaglinide in IVM, IVF, and follicle growth culture medium significantly improved in vitro mice oocyte maturation, fertilization, embryo cleavage, and follicle growth rates, probably by elevating intracellular calcium concentrations (Kalehoei & Azadbakht, 2017; Awadh *et al.*, 2019; 2020). The study of Mahdi & AL-Hady (2021) showed that combination treatments with metformin (500 mg/kg) and repaglinide (4 mg/kg) in diabetic rats had a positive effect on sperm parameters such as motility and viability. Kumar *et al.* (2008) demonstrated that high doses of repaglinide (6.5 mM) and similar compounds, depending on dose and time, caused a large increase in intracellular Ca^2+^ and were toxic to human sperm. The study by Mizuno *et al.* (2012) indicated that chemotactic properties of sperm significantly decreased at repaglinide concentrations above 100 µM (> 100 µM) with no significant changes in sperm swimming. Our findings showed that repaglinide at concentrations of 100 nM and 1µM increased sperm motility and hyperactivation in specimens from normozoospermic and asthenozoospermic men, although higher progressive motility in the normozoospermic group was observed after treatment with 1µM of repaglinide; in the asthenozoospermic group, there was no significant difference in progressive motility between controls and specimens treated with repaglinide; enhanced total motility in terms of increased non-progressive motility and best percent hyperactivity were obtained at a dose of 100 nM of repaglinide.

Samplaski *et al.* (2015) indicated a strong relationship between viability rate and DNA fragmentation. Reduction of sperm viability was associated with high sperm DNA damage; in other words, a high viability rate was correlated with a low rate of sperm DNA fragmentation. In this regard, our study showed that in both normozoospermic and asthenozoospermic groups, higher sperm viability rate and lower percentages of DNA fragmentation were observed in specimens treated with repaglinide compared with controls. The study by Li *et al*. (2016) demonstrated that repaglinide with antioxidant activity had a protective effect against kidney and renal tubular oxidative injury induced by cyclosporine. In the present study, antioxidant properties apparently linked to repaglinide preserved sperm viability and reduced sperm DNA fragmentation during in vitro induction.

## CONCLUSION

Our experimental study found that repaglinide can improve in vitro human sperm motility, hyperactivity, viability, and DNA integrity in both normozoospermic and asthenozoospermic men. This finding might be important in the preparation of sperm to improve IUI, IVF, and ICSI outcomes for selected couples.
